# Foretinib Alleviates Osteoblast Senescence and Protects Against Bone Loss in Ovariectomized Mice by Promoting Osteoblast Differentiation

**DOI:** 10.3390/cells14241945

**Published:** 2025-12-08

**Authors:** Jiin Oh, Jueun Lee, Eok-Cheon Kim, Jae-Ryoung Kim, Hyunil Ha, Taesoo Kim, Kyunghee Lee, Daewon Jeong

**Affiliations:** 1 Laboratory of Bone Metabolism and Control, Department of Microbiology, Yeungnam University College of Medicine, Daegu 42415, Republic of Korea; jjvmf@ynu.ac.kr (J.O.); wndms4864@ynu.ac.kr (J.L.); 2 Department of Biochemistry and Molecular Biology, Senotherapy-Based Metabolic Disease Control Research Center, Yeungnam University College of Medicine, Daegu 42415, Republic of Korea; skybill7@gmail.com (E.-C.K.); kimjr@med.yu.ac.kr (J.-R.K.); 3 Herbal Medicine Research Division, Korea Institute of Oriental Medicine, Daejeon 34054, Republic of Korea; hyunil74@kiom.re.kr (H.H.); xotn91@kiom.re.kr (T.K.)

**Keywords:** osteoblast senescence, osteoblast differentiation, SASP, foretinib, osteoporosis

## Abstract

Osteoporosis is a major global health challenge, causing millions of fragility fractures each year and imposing an escalating socioeconomic burden worldwide. Despite advances with antiresorptive and anabolic therapies, substantial residual fracture risk persists, and targeting aging biology may yield disease modifying benefits beyond current standards of care. Senescent cells secrete senescence-associated secretory phenotype (SASP) factors, which impair osteoblast differentiation and contribute to bone loss. We investigated foretinib, a quinoline-based multi-tyrosine kinase inhibitor, as a potential anti-aging agent in osteoblast lineage cells. Foretinib inhibited doxorubicin-induced senescence in osteoblast progenitors via the p53/p21 and p16 pathways and reduced the expression of osteogenesis-inhibiting SASP factors, including CCL2, interleukin (IL)-1α, IL-1β, and IL-6. As a result, foretinib restored the impaired osteogenic differentiation of aged osteoblasts to near-normal levels in vitro. In ovariectomized, estrogen-deficient mice, foretinib significantly reduced trabecular and cortical bone loss by enhancing in vivo osteoblast differentiation, as shown by histological analysis and micro-computed tomography of femoral bone. These results suggest that foretinib alleviates osteoblast senescence and enhances osteogenic differentiation, supporting its promise as a therapeutic candidate for postmenopausal osteoporosis.

## 1. Introduction

Aging is accompanied by progressive functional decline across tissues, to which cellular senescence is a major contributor. Senescence is characterized by irreversible cell cycle arrest, enlarged flattened morphology, and a distinct molecular program [1,2]. Two canonical tumor suppressor pathways enforce senescence-associated arrest: the p53/p21 pathway and the p16/retinoblastoma protein (Rb) pathway. Cellular stressors such as DNA damage activate p53, which induces p21, inhibiting cyclin-dependent kinase (CDK) activity and blocking cell cycle progression. In parallel, p16 inhibits CDK4/6 and prevents Rb phosphorylation, and the resulting hypophosphorylated Rb suppresses E2F-dependent transcription to reinforce arrest [1,3,4].

Senescent cells secrete a senescence-associated secretory phenotype (SASP) comprising tumor necrosis factor-α (TNF-α), interleukin (IL)-1α, IL-1β, IL-6, and C-C motif chemokine ligand 2 (CCL2). These factors act in autocrine and paracrine fashions to propagate senescence and modulate neighboring cell behavior, often sustaining chronic low-grade inflammation [3,5,6,7]. In the skeletal microenvironment, the SASP impairs osteoblast differentiation while promoting osteoclastogenesis, thereby shifting remodeling toward bone loss. Experimental suppression of SASP signaling in bone lineage cells restores osteogenic capacity and improves skeletal homeostasis [8,9,10,11,12].

Postmenopausal osteoporosis is characterized by reduced bone mass, deterioration of microarchitecture, and heightened fracture risk. Estrogen deficiency amplifies inflammatory signaling and receptor activator of nuclear factor kappa B ligand activity, driving osteoclast-mediated resorption over osteoblast-driven formation [13,14]. The ovariectomized mouse replicates key features of estrogen deficiency–induced bone loss and is widely used to assess interventions that address remodeling and quality in addition to density [15]. These considerations underscore the therapeutic need for approaches that modulate aging biology, including cellular senescence and the SASP, to rebalance bone remodeling and restore bone quality.

Foretinib, a small-molecule multi-kinase inhibitor developed for oncology, targets mesenchymal–epithelial transition factor (MET) and vascular endothelial growth factor receptor pathways, which intersect with stromal and inflammatory signaling relevant to bone remodeling [16,17]. Moreover, our group has proposed and demonstrated with a related multi-kinase inhibitor that suppression of p53/p21 and p16/Rb senescence signaling, attenuation of the SASP, enhancement of osteoblast differentiation, and inhibition of osteoclastogenesis are feasible strategies to counter skeletal aging—providing proof of concept for repurposing this drug class in bone [7,18]. This prior work supports the rationale for evaluating foretinib in skeletal contexts.

Based on these considerations, we tested the hypothesis that foretinib functions as an anti-aging osteoanabolic agent by suppressing cellular senescence and the SASP in osteolineage cells. We demonstrate that foretinib reduces senescence-associated signaling, restores osteoblast differentiation, and therapeutically reverses ovariectomy-induced bone loss, thereby establishing preclinical value for foretinib as an intervention targeting aging biology to counter postmenopausal estrogen deficiency osteoporosis.

## 2. Materials and Methods

### 2.1. Cell Culture and Osteoblast Differentiation

Primary osteoblast progenitors were isolated from the calvaria of newborn C57BL/6 mice (SAMTAKO Bio Korea, Osan, Republic of Korea) by sequential digestion with 0.25% trypsin (Cytiva, Marlborough, MA, USA) for 10 min at 37 °C and 0.1% collagenase II (Worthington Biochemical, Lakewood, NJ, USA) for 30–60 min at 37 °C with gentle rocking [19]. Cells were centrifuged (2000 rpm, 5 min), resuspended in alpha minimum essential medium (α-MEM; Cytiva, Cat. No. SH30265.01) supplemented with 10% (*v*/*v*) fetal bovine serum (FBS; Cytiva) and 1× antibiotic–antimycotic (Thermo Fisher Scientific, Waltham, MA, USA) and maintained at 37 °C in 5% CO_2_. For osteogenic differentiation, osteoblast progenitors were seeded at 2.0 × 10^4^ cells/well (48-well plates) and cultured in osteogenic medium (α-MEM, 10% FBS, 1× antibiotic–antimycotic) containing 4 mM sodium phosphate (Biosolution, Seoul, Republic of Korea); medium was refreshed every 2 days. At the indicated times, cells were washed with phosphate-buffered saline (PBS), fixed in ice-cold 70% ethanol for 30 min and stained with 40 mM Alizarin Red S (ARS; pH 4.2; Sigma-Aldrich, St. Louis, MO, USA) for 30 min at room temperature. For quantification, bound ARS was solubilized with 10% cetylpyridinium chloride (pH 7.0; Sigma-Aldrich) for 15 min, and absorbance was measured at 550 nm (Thermo Fisher Scientific).

### 2.2. Induction of Cellular Senescence and SA-β-Gal Staining

Doxorubicin (DOX)-induced senescence was generated by exposing osteoblast progenitors (2.0 × 10^4^ cells/well, 48-well plates) to 0.2 μM DOX (Sigma-Aldrich) for 4 h, followed by culture in the indicated media [PBS vehicle or foretinib (100 nM)]. For SA-β-gal staining [7], cells were washed with PBS, fixed with 3.7% paraformaldehyde (30 min, room temperature), and incubated in SA-β-gal staining solution (0.2 M citric acid/phosphate, pH 6.0; 5 mM potassium ferrocyanide; 5 mM potassium ferricyanide; 150 mM NaCl; 2 mM MgCl_2_; and 0.5 mg/mL X-gal in dimethyl sulfoxide [DMSO]) at 37 °C for 16 h. Images were collected from four random fields per well, and SA-β-gal–positive cells were expressed as a percentage of total cells.

### 2.3. Western Blot Analysis

Cells were lysed in RIPA buffer (Thermo Fisher Scientific) supplemented with a protease inhibitor cocktail (Roche, Basel, Switzerland) and phosphatase inhibitors (β-glycerophosphate, NaF, and Na_3_VO_4_; Sigma-Aldrich). Lysates were cleared by centrifugation (13,000× *g*, 10 min, 4 °C), protein concentrations determined, and 20–30 μg loaded per lane on 10–12% SDS–PAGE. Proteins were transferred to nitrocellulose membranes (Cytiva), blocked in Tris-buffered saline with Tween 20 (TBST) containing 0.2% (*w*/*v*) I-BLOCK (Thermo Fisher Scientific) for 1 h, and probed overnight at 4 °C with primary antibodies: p53, p16, and runt-related transcription factor 2 (RUNX2) (Cell Signaling Technology, Danvers, MA, USA); p21, β-catenin, alkaline phosphatase (ALP), Fra-1, and β-actin (Santa Cruz, Dallas, TX, USA); and osteopontin (OPN) and osterix (OSX) (Abcam, Cambridge, UK). After TBST washes, membranes were incubated with horseradish peroxidase-conjugated goat anti-mouse or anti-rabbit secondary antibodies (room temperature, 2 h) and developed using enhanced chemiluminescence (AbFRONTIER, Seoul, Republic of Korea).

### 2.4. Enzyme-Linked Immunosorbent Assay (ELISA)

For SASP quantification, osteogenic culture media were replaced every two days. To measure accumulated secretion, conditioned media corresponding to the interval between days 4 and 6 after FTB exposure were collected (centrifugation 1400× *g*, 10 min), aliquoted, and stored at −80 °C until analysis. SASP cytokines (IL-1α, IL-1β, IL-6, and CCL2) were quantified using DuoSet ELISA kits (R&D Systems, Minneapolis, MN, USA) according to the manufacturer’s instructions, and absorbance was read on a microplate reader (Thermo Fisher Scientific). To account for differences in cell content, ELISA values were normalized to total cellular protein from the corresponding cultures (quantified after media collection), and data are reported as normalized SASP levels.

### 2.5. Conditioned Media Assay

For paracrine assays, conditioned media were collected on day 6 from control, DOX, or DOX + foretinib (FTB) cultures, centrifuged, and mixed 1:1 with fresh osteogenic medium containing 4 mM inorganic phosphate. Osteoblast progenitors were then differentiated for an additional 11 days, followed by ARS staining and quantification as described in Section 2.1.

### 2.6. Ovariectomy and Therapeutic Foretinib Dosing

Female C57BL/6 mice (9 weeks old; SAMTAKO) were acclimated for 1 week. At 10 weeks, mice were anesthetized with 1.25% avertin (Sigma-Aldrich) via intraperitoneal injection and subjected to bilateral ovariectomy (OVX) to induce estrogen deficiency–related bone loss. Sham-operated mice underwent identical procedures without ovary removal. Animals were randomized into three groups: Sham + vehicle, OVX + vehicle, and OVX + FTB (n = 8/group). For the therapeutic regimen, FTB (0.315 mg/kg in 0.1% DMSO/PBS) or vehicle was administered intraperitoneally every 2 days for 8 weeks, beginning 8 weeks post-OVX. Mice were euthanized at 26 weeks of age, and femurs and tibiae were harvested, fixed in 3.7% formaldehyde for 3 days, and stored in 70% ethanol prior to μCT and histological analyses. All animal procedures complied with institutional guidelines and were approved by the Yeungnam University Medical Center (YUMC-AEC2023-027).

### 2.7. Dual-Energy X-Ray Absorptiometry (DXA) and Micro-Computed Tomography (μCT) Analysis

After anesthesia, whole-body DXA was performed to quantify bone mineral density (BMD) and fat percentage using a cabinet DXA analyzer (iNSiGHT VET DXA, Osteosys, Seoul, Republic of Korea), as described previously [20]. Ex vivo μCT of femurs was conducted using a Skyscan1276 system (Skyscan, Aartselaar, Belgium) equipped with a 0.5 mm Al filter (typical settings: 70 kV, 57 μA). Images were reconstructed with NRecon (v1.7.42, Bruker). Trabecular microarchitecture was evaluated at the distal femur (region beginning 80 μm proximal to the growth plate, spanning 200 cross-sections), and cortical parameters were assessed at the mid-diaphysis. Quantified indices included trabecular BMD, trabecular bone volume per tissue volume (BV/TV), trabecular number (Tb.N), trabecular separation (Tb.Sp), and cortical BMD, BV/TV, cortical thickness (Ct.Th), and cortical area (Ct.Ar). Analyses were performed using CTAn (v1.20.3.0, Bruker).

### 2.8. Histomorphometry Analysis

Fixed femurs were decalcified in 14% neutral-buffered EDTA (Sigma-Aldrich) for 3 weeks and embedded in paraffin. Sagittal sections (5 μm) were stained with H&E to assess osteoblasts. Osteoblast parameters included N.Ob/B.Pm (osteoblast number per bone perimeter) and Ob.S/BS (osteoblast surface per bone surface). Histomorphometric terminology and units followed ASBMR recommendations, and analyses were performed blinded using a semi-automated system (OsteoMeasure; OsteoMetrics, Decatur, GA, USA).

### 2.9. Statistical Analysis

Data are presented as mean ± SD when parametric assumptions are met; otherwise, distribution-appropriate summaries are provided. Normality was assessed using the Shapiro–Wilk test and variance equality using Levene’s test. For two-group comparisons, Student’s two-tailed *t*-test was applied when assumptions held; for comparisons among three or more groups, one-way ANOVA with suitable post hoc comparisons was used. When assumptions were not met, assumption-robust methods were employed. Significance was set at *p* < 0.05. Analyses were performed in GraphPad Prism (GraphPad 8.0.2 Software, San Diego, CA, USA). Significance notation in figures: ns, not significant; * *p* < 0.05; ^#^ *p* < 0.01.

## 3. Results

### 3.1. Foretinib Suppresses Osteoblast Progenitor Senescence

**Figure 1:**
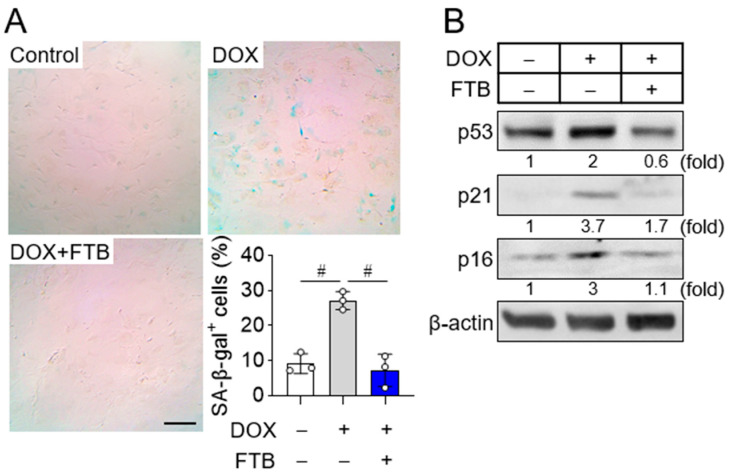
Foretinib suppresses osteoblast senescence. (**A**) Assessment of senescence. Osteoblast progenitors were exposed to 0.2 μM doxorubicin (DOX) for 4 h to induce senescence, followed by culture with 100 nM foretinib (FTB) or DMSO (Control) in α-MEM for 6 days. Senescent cells were detected by SA-β-gal staining, and the proportion of SA-β-gal–positive (SA-β-gal^+^) cells was quantified from four randomly selected fields per sample in triplicate cultures. Data are shown as mean ± SD from one of three independent experiments (triplicate per condition). ^#^ *p* < 0.01. Scale bar, 100 μm. (**B**) Expression of senescence markers. DOX-induced senescent osteoblast progenitors were treated with 100 nM FTB for 24 h, lysed, and analyzed by Western blotting for p53, p21, and p16, with β-actin as a loading control. Protein levels were quantified as fold change relative to untreated controls.

After age 50, bone resorption surpasses bone formation, resulting in osteoporosis and heightened fracture risk. Anti-aging agents are considered promising for osteoporosis prevention. To this end, we screened potential anti-aging compounds—including previously reported drugs and natural products—using senescent fibroblasts induced by doxorubicin, a topoisomerase II inhibitor [21,22]. In this preliminary screen, foretinib, a multi-tyrosine kinase inhibitor with reported anticancer activity in ovarian and acute myeloid leukemia [23,24], was the most effective anti-aging candidate. The non-cytotoxic dose of foretinib in osteoblast progenitors was established as 100 nM (Supplementary Figure S1). Compared with controls, foretinib markedly reduced the number of doxorubicin-induced senescent osteoblast progenitors (Figure 1A), as assessed by enlarged, flattened cell morphology and senescence-associated β-galactosidase (SA-β-gal) staining, a widely used marker of senescence in cell culture and mammalian tissues [25]. In addition, this finding was confirmed in a prolonged culture–induced aging model across multiple passages. In senescent cells at passage 6 (P6), foretinib significantly reduced SA-β-gal–positive cells (Supplementary Figure S2). Cellular senescence involves irreversible cell cycle arrest through activation of the p53/p21WAF1/CIP1 and/or p16INK4A/Rb tumor suppressor pathways, which block uncontrolled proliferation [1,3,4]. Foretinib normalized the doxorubicin-induced upregulation of p53, p21, and p16 in osteoblast progenitors to control levels (Figure 1B). Collectively, these findings indicate that foretinib attenuates osteoblast progenitor senescence by modulating the p53/p21 and p16/Rb pathways.

### 3.2. Foretinib Promotes Osteoblast Differentiation by Reducing Osteogenic Inhibitor SASP Factors

**Figure 2:**
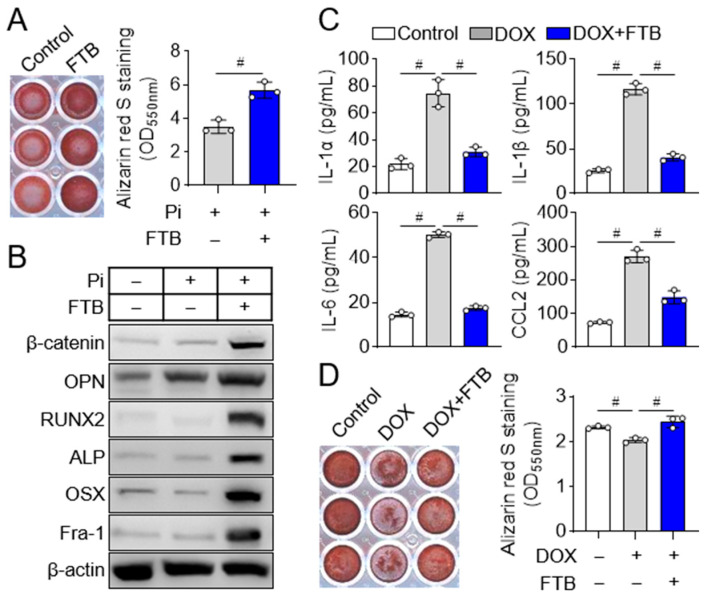
Foretinib stimulates osteoblast differentiation. (**A**) Alizarin Red S staining and quantification of mineralized nodules. Senescent osteoblast progenitors induced with doxorubicin (DOX) were cultured in an osteogenic medium containing 4 mM inorganic phosphate and 100 nM foretinib (FTB) for 11 days. Mineralized nodules were visualized by Alizarin Red S staining (**left**), and calcium deposition was quantified by dye solubilization with cetylpyridinium chloride followed by absorbance measurement at 550 nm (**right**). Data represent mean ± SD from one of three independent experiments (triplicate per condition). ^#^ *p* < 0.01. (**B**) Expression of osteogenic markers. Senescent osteoblast progenitors were treated as indicated and lysed on day 8 of differentiation. Immunoblots were probed for β-catenin, osteopontin (OPN), RUNX2, alkaline phosphatase (ALP), osterix (OSX), and Fra-1, with β-actin as a loading control. (**C**) SASP factor analysis. Non-senescent (control) or senescent osteoblast progenitors were treated with or without FTB for 6 days. Culture media were replaced every 2 days; for ELISA, culture media corresponding to the interval between days 4 and 6 after FTB exposure were collected. Levels of IL-1α, IL-1β, IL-6, and CCL2 were quantified by ELISA and normalized to total cellular protein. Data represent mean ± SD from one of three independent experiments (triplicate per condition). ^#^ *p* < 0.01. (**D**) Conditioned media assay for osteogenic differentiation. Media collected on day 6 from cultures under the indicated conditions (Control, DOX, DOX + FTB) were mixed 1:1 with fresh medium to generate conditioned media. Osteoblast progenitors were then differentiated for 11 days in conditioned media containing 4 mM inorganic phosphate. Differentiation was assessed by ARS staining of mineralized nodules; plates were imaged, dye solubilized with cetylpyridinium chloride, and absorbance at 550 nm measured to quantify calcium deposition. Data represent mean ± SD from one of three independent experiments (triplicate per condition). ^#^ *p* < 0.01.

To examine the role of foretinib in osteoblast function, we induced senescence in osteoblast progenitors with doxorubicin and differentiated them in osteogenic medium supplemented with 4 mM inorganic phosphate. Under these conditions, foretinib (FTB) significantly enhanced osteoblast differentiation relative to control, as shown by increased mineralized nodule formation visualized with Alizarin Red S staining and quantified by dye solubilization with absorbance at 550 nm (Figure 2A). Notably, in a prolonged culture–induced aging model, senescent osteoblast progenitors at passage 6 (P6) again showed markedly enhanced osteoblast differentiation with foretinib, as evidenced by increased mineralized nodule formation relative to control (Supplementary Figure S3). In agreement, immunoblotting revealed elevated expression of osteogenic markers in FTB-treated senescent cells, including β-catenin, osteopontin (OPN), runt-related transcription factor 2 (RUNX2), alkaline phosphatase (ALP), osterix (OSX), and Fra-1 (Figure 2B).

Because senescent cells release senescence-associated secretory phenotype (SASP) factors that impair osteoblast differentiation [8], we quantified representative SASP cytokines in culture media. ELISA showed that DOX-induced senescence increased IL-1α, IL-1β, IL-6, and CCL2, whereas FTB treatment markedly reduced their levels (Figure 2C). To assess the functional impact of SASP on osteogenesis, we performed a conditioned media assay. Media collected on day 6 from control, DOX, and DOX + FTB cultures were mixed 1:1 with fresh osteogenic medium, and osteoblast progenitors were differentiated for an additional 11 days in the presence of 4 mM inorganic phosphate. Conditioned media from DOX cultures suppressed mineralized nodule formation, whereas media from DOX + FTB cultures alleviated this effect and restored osteogenic capacity (Figure 2D). Together, these findings demonstrate that foretinib enhances osteoblast differentiation in senescent progenitors, at least in part by suppressing SASP factor production and its inhibitory paracrine influence.

### 3.3. Foretinib Recovers Bone Loss in Ovariectomized Mice by Enhancing Osteoblast Differentiation

**Figure 3:**
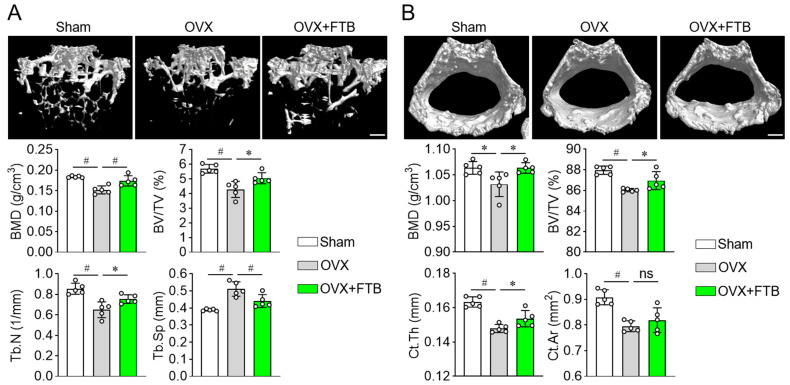
Foretinib therapeutically reverses ovariectomy (OVX)-induced bone loss in vivo. (**A**) Trabecular bone µCT. Representative 3D reconstructions and quantitative analyses of the trabecular compartment in Sham, OVX, and OVX + FTB groups. Therapeutic foretinib (FTB; 0.315 mg/kg, 500 nM) was administered beginning 8 weeks after OVX and continued for 8 weeks. Quantified indices include bone mineral density (BMD), bone volume fraction (BV/TV), trabecular number (Tb.N), and trabecular separation (Tb.Sp). (**B**) Cortical bone µCT. Representative images and quantitative analyses of the cortical compartment in Sham, OVX, and OVX + FTB groups. Parameters include cortical BMD, BV/TV, cortical thickness (Ct.Th), and cortical area (Ct.Ar). Data are expressed as mean ± SD (n = 5). ns, not significant; * *p* < 0.05; ^#^ *p* < 0.01. Scale bar, 0.5 mm.

Because foretinib (FTB) promotes osteoblast differentiation in vitro, we next assessed its osteoanabolic activity in an ovariectomy (OVX)–induced osteoporosis model using two dosing strategies. In the prevention regimen, FTB was administered immediately after OVX for 8 weeks but failed to rescue OVX-induced bone loss; dual-energy X-ray absorptiometry (DXA) and micro-computed tomography (µCT) revealed no significant improvements compared with OVX controls. In the treatment regimen, FTB was initiated 8 weeks after OVX—when osteopenia was established—and continued for an additional 8 weeks. As expected, OVX mice gained more body weight than Sham (Supplementary Figure S4A), consistent with a menopause-like phenotype, and FTB attenuated this OVX-associated weight gain. DXA analysis further demonstrated that therapeutic FTB increased whole-body BMD while reducing fat percentage (Supplementary Figure S4B). In parallel, µCT revealed marked restoration of trabecular microarchitecture with FTB, including higher BMD, increased bone volume fraction (BV/TV), greater trabecular number (Tb.N), and reduced trabecular separation (Tb.Sp) (Figure 3A). Cortical bone was also improved, with increases in cortical BMD, BV/TV, cortical thickness (Ct.Th), and cortical area (Ct.Ar) in FTB-treated OVX mice (Figure 3B). Together, these results indicate that FTB provides a therapeutic—rather than preventive—benefit in OVX-induced bone loss, restoring bone mass and microarchitecture once deterioration has already occurred.

**Figure 4:**
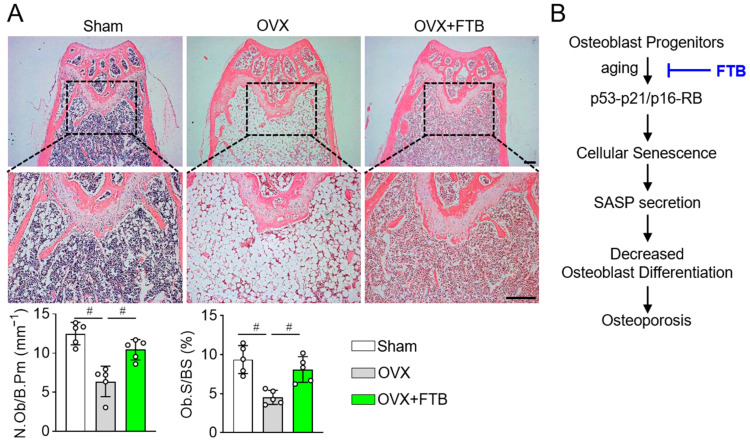
Foretinib rescues osteoblast deficits in ovariectomized mice. (**A**) Representative H&E-stained femoral sections from Sham, OVX, and OVX + FTB groups with enlarged insets. Therapeutic foretinib (FTB; 0.315 mg/kg) was administered 8 weeks after OVX and continued for 8 weeks (same regimen as in Figure 3). Histomorphometric quantification of osteoblast indices: N.Ob/B.Pm, osteoblast number per bone perimeter; Ob.S/BS, osteoblast surface per bone surface. Data are expressed as mean ± SD (n = 5). ^#^ *p* < 0.01. Scale bar, 250 μm. (**B**) Schematic of the FTB-mediated anti-osteoporotic mechanism.

Consistent with the improvements observed in trabecular and cortical compartments (Figure 3), H&E staining with histomorphometric analysis revealed that osteoblast number per bone perimeter (N.Ob/B.Pm) and osteoblast surface per bone surface (Ob.S/BS) were significantly increased in OVX + FTB mice compared with OVX controls, approaching values seen in Sham animals (Figure 4A). These findings support a model in which foretinib mitigates OVX-induced bone loss primarily by enhancing osteoblast activity.

## 4. Discussion

We began with a phenotypic screen for anti-aging activity and identified foretinib as a candidate that suppresses doxorubicin (DOX)-induced senescence in osteoblast progenitors at a non-cytotoxic concentration (100 nM). Under these conditions, foretinib reduced the proportion of SA-β-gal–positive cells and normalized activation of the canonical p53/p21 and p16/Rb senescence pathways, providing a mechanistic basis for its downstream osteogenic effects. Together, our findings suggest that foretinib attenuates postmenopausal osteoporosis by suppressing cellular senescence through the p53–p21/p16–Rb pathway axes, decreasing SASP secretion, and restoring osteogenic capacity (Figure 4B). These results support the concept that targeting cellular senescence in osteolineage cells represents a viable strategy to mitigate age-associated skeletal decline. In addition, our findings in the doxorubicin-induced model align with canonical senescence features—stable cell-cycle arrest, SASP upregulation, and SA-β-gal activity—consistent with established literature, supporting their generalizability [26,27,28]. Although triggering stimuli differ (acute genotoxic stress versus telomere-driven replicative stress), both models converge on similar downstream programs, including persistent DNA damage response signaling and p53–p21/p16–Rb pathway activation. This convergence provides a mechanistic rationale for expecting qualitatively similar outcomes in prolonged-culture (replicative) senescence, particularly in inflammatory signaling, chromatin remodeling, and metabolic reprogramming. We acknowledge that model-specific kinetics and pathway amplitudes may vary; therefore, targeted validation in prolonged-culture systems represents an important avenue for future work.

To establish the connection between osteoblast differentiation and SASP production, we examined whether senescent osteoblast progenitors release SASP factors that impair osteogenesis [5,8]. DOX-induced senescence elevated secretion of IL-1α, IL-1β, IL-6, and CCL2, whereas foretinib reduced these cytokines and concomitantly enhanced mineralization and osteogenic marker expression. Moreover, conditioned media assays directly linked SASP suppression to improved osteogenesis: media from DOX cultures inhibited mineralized nodule formation, while media from DOX + foretinib (FTB) cultures alleviated this inhibition and restored differentiation. Together, these findings demonstrate that FTB not only dampens intrinsic senescence programs but also remodels the paracrine and/or autocrine environment to support osteoblast differentiation.

In vitro, concurrent DOX and foretinib treatment was designed to model the onset of senescence and therefore indicates a preventive effect, whereby foretinib limits establishment of the senescent phenotype. By contrast, in the ovariectomized (OVX) model, foretinib administration was initiated only after osteopenia had been verified by DXA and µCT, demonstrating therapeutic efficacy in an established bone loss context. Together, these data support a dual action of foretinib, preventing senescence associated dysfunction under prosenescent stress and treating bone loss driven by estrogen deficiency. In vivo, the timing of intervention proved critical. Foretinib failed to prevent bone loss when administered immediately after OVX for 8 weeks, but it restored bone when treatment was initiated 8 weeks post-OVX (therapeutic regimen), after osteopenia or osteoporosis had developed. Under therapeutic dosing, whole-body DXA revealed improved BMD and reduced adiposity, and µCT demonstrated recovery of both trabecular and cortical compartments—elevated BMD, increased BV/TV and Tb.N, reduced Tb.Sp, and improved cortical indices (BMD, BV/TV, Ct.Th, Ct.Ar). Histological analysis reinforced this osteoanabolic profile, showing greater N.Ob/B.Pm and Ob.S/BS, thereby linking cellular activity to structural improvements. These findings indicate that the primary utility of foretinib lies in therapy—reversing established deficits—rather than prevention in the immediate post-OVX phase, consistent with a mechanism focused on alleviating senescence-associated dysfunction rather than blocking its initiation. Moreover, the concordance across DXA, µCT, and histomorphometry supports a model in which foretinib relieves senescence-driven impairments in osteoblast function, enabling both trabecular and cortical rebuilding. The increases in osteoblast number and surface, together with restored microarchitecture, suggest that the drug’s dominant effect is to normalize osteoblast lineage activity in a senescent context rather than directly suppress resorption. This interpretation aligns with the in vitro data linking SASP reduction to enhanced osteogenesis and with the in vivo pattern of therapeutic but not preventive efficacy. Future directions include combination strategies with anti-resorptives (e.g., bisphosphonates, denosumab) [12,29,30,31] and direct comparisons with established anabolic agents (e.g., teriparatide, romosozumab) [32,33,34].

Clinically, foretinib is administered at high oncology doses—either 80 mg daily [16] or 240 mg daily for 5 days followed by 9 days off [17,35]—where adverse events such as hypertension, fatigue, gastrointestinal disturbance, elevated liver enzymes, and occasional thrombosis are common [16,35]. In contrast, our study employed much lower exposures (0.315 mg/kg in mice and 100 nM in vitro) aimed at reducing senescence/SASP and enhancing osteoblast differentiation rather than eliciting cytotoxic effects. By simple body-surface-area conversion, 0.315 mg/kg in mice corresponds to ~0.026 mg/kg in humans (approximately 1–2 mg/day for a 70 kg adult), well below oncology dosing. Such reduced dosing is likely to be more tolerable, compatible with long-term use (e.g., in postmenopausal women with established osteoporosis), and afford a wider safety margin for combination with antiresorptive therapies.

Our findings were generated primarily in an ovariectomized osteoporosis model, which limits generalizability; future studies should assess foretinib’s capacity to restore bone in natural aging and in the accelerated aging Zmpste24 model, with particular emphasis on bone healing and regeneration. To enhance translational relevance, investigations should incorporate human-derived osteoblast progenitors and advanced in vitro bone microenvironment models, with outcomes benchmarked against non-senescent baselines. Dose and timing optimization, along with longer-term readouts including fracture healing, are needed to define safety margins and durability of effect.

In summary, foretinib suppresses osteoblast progenitor senescence, alleviates SASP-mediated inhibition, and restores osteoblast-driven bone formation in established osteopenia, resulting in trabecular and cortical recovery in OVX mice under a therapeutic regimen. These findings position senescence-targeted osteoanabolism as a promising strategy to reverse—rather than merely prevent—osteoporotic bone loss.

## 5. Conclusions

Foretinib is clinically associated with a range of adverse effects across cardiovascular, hepatic, and gastrointestinal systems, with occasional hematologic and thrombotic events. The study was not powered for safety, so careful clinical monitoring and dose optimization for bone indications are needed, and longer term and fracture healing studies should define skeletal safety and clarify the overall risk and benefit.

Osteoporosis remains the most prevalent aging-related skeletal disorder. Here, we applied foretinib to aged osteolineage cells and a postmenopausal osteoporosis mouse model, confirming its therapeutic efficacy. Our results demonstrate that foretinib attenuates senescence in osteoblast progenitors and restores their differentiation potential through SASP suppression. Collectively, these findings identify foretinib as a potential therapeutic candidate for postmenopausal osteoporosis.

## Data Availability

In this study, the datasets are available on request to the corresponding author.

## Supplementary Materials

The following supporting information can be downloaded at: https://www.mdpi.com/article/10.3390/cells14241945/s1. Figure S1. Cytotoxicity of foretinib in senescent osteoblast progenitors. Osteoblast progenitors were treated with doxorubicin for 4 h, washed with culture medium, and then cultured with phosphate-buffered saline (PBS; Control) or foretinib (FTB, 100 nM) for 4 (d4) or 6 days (d6). Cell viability was assessed by the MTT [3-(4,5-dimethylthiazol-2-yl)-2,5-diphenyltetrazolium bromide] assay and expressed as a percentage of the control. The purple formazan crystals were dissolved in dimethyl sulfoxide, and absorbance was measured at 570 nm. Data are presented as mean ± SD from triplicate experiments; Figure S2. Foretinib suppresses osteoblast senescence. Evaluation of senescence. Osteoblast progenitors at passage 3 (P3; control) and senescent cells at passage 6 (P6; SEN) were cultured in α-MEM supplemented with either 100 nM foretinib (FTB) or DMSO (Control) for 6 days. Senescence was assessed by SA-β-gal staining, and the fraction of SA-β-gal–positive (SA-β-gal^+^) cells was determined from four randomly selected fields per sample across triplicate cultures. Data are presented as mean ± SD from one of three independent experiments (triplicate per condition). ^#^ *p* < 0.01. Scale bar, 100 μm; Figure S3. Foretinib promotes osteoblast differentiation. Alizarin Red S staining and quantification of mineralized nodules. Senescent osteoblast progenitors at passage 6 (P6; SEN) were cultured for 12 days in an osteogenic medium containing ascorbic acid (50 µg/mL), β-glycerophosphate (10 mM), and bone morphogenetic protein-2 (BMP-2; 200 ng/mL), with or without 100 nM foretinib (FTB). Mineralized nodules were visualized by Alizarin Red S staining (**left**), and calcium deposition was quantified by dye solubilization with cetylpyridinium chloride followed by absorbance measurement at 550 nm (**right**). Data represent mean ± SD from one of three independent experiments (triplicate per condition). * *p* < 0.05; Figure S4. Body composition analysis. (**A**) Body weight profile. Ten-week-old female mice underwent sham surgery or ovariectomy (OVX) and were assigned to three groups: sham-operated (Sham), OVX, and OVX treated with foretinib (OVX+FTB). Eight weeks after surgery, foretinib (FTB; 0.315 mg/kg) or vehicle was administered intraperitoneally every 2 days for 8 weeks. Body weight was measured weekly for 16 weeks. The arrowhead indicates the onset of FTB administration. (**B**) DXA analysis. Mice were euthanized at 26 weeks of age, and bone mineral density (BMD) and fat mass were analyzed using a high-resolution DXA scanner. In the color composite image, fat and lean tissues are shown in red and green, respectively. Data are presented as mean ± SD (n = 5 per group). ^#^ *p* < 0.01.
