# Supplementary material for: Foretinib Alleviates Osteoblast Senescence and Protects Against Bone Loss in Ovariectomized Mice by Promoting Osteoblast Differentiation
Source: Cells. 2025 Dec 8;14(24):1945. doi: 10.3390/cells14241945 (PMC12731566; doi:10.3390/cells14241945)

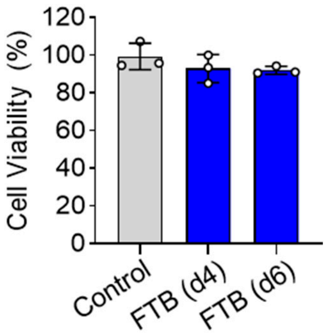

Figure S1. Cytotoxicity of foretinib in senescent osteoblast progenitors. Osteoblast progenitors were treated with doxorubicin for 4 h, washed with culture medium, and then cultured with phosphate-buffered saline (PBS; Control) or foretinib (FTB, 100 nM) for 4 (d4) or 6 days (d6). Cell viability was assessed by the MTT [3-(4,5-dimethylthiazol-2-yl)-2,5-diphenyltetrazolium bromide] assay and expressed as a percentage of the control. The purple formazan crystals were dissolved in dimethyl sulfoxide, and absorbance was measured at 570 nm. Data are presented as mean ± SD from triplicate experiments.

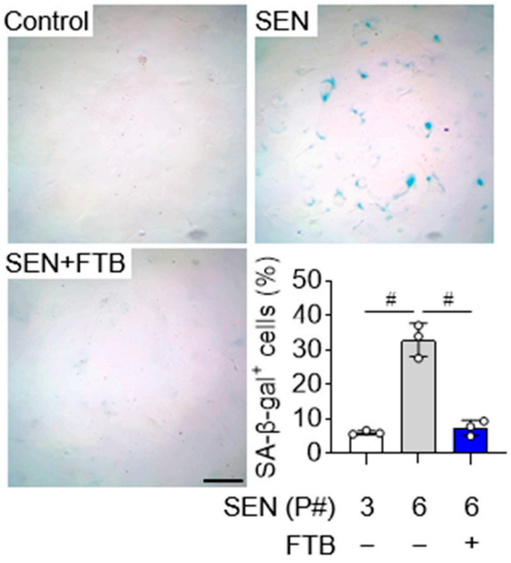

Figure S2. Foretinib suppresses osteoblast senescence. Evaluation of senescence. Osteoblast progenitors at passage 3 (P3; control) and senescent cells at passage 6 (P6; SEN) were cultured in α-MEM supplemented with either 100 nM foretinib (FTB) or DMSO (Control) for 6 days. Senescence was assessed by SA-β-gal staining, and the fraction of SA-β-gal–positive (SA-β-gal$^+$) cells was determined from four randomly selected fields per sample across triplicate cultures. Data are presented as mean ± SD from one of three independent experiments (triplicate per condition). $^{\#}p < 0.01$. Scale bar, 100 μm.

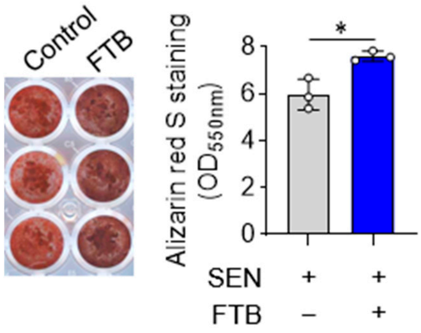

Figure S3. Foretinib promotes osteoblast differentiation. Alizarin Red S staining and quantification of mineralized nodules. Senescent osteoblast progenitors at passage 6 (P6; SEN) were cultured for 12 days in an osteogenic medium containing ascorbic acid (50 µg/ml), β-glycerophosphate (10 mM), and bone morphogenetic protein-2 (BMP-2; 200 ng/ml), with or without 100 nM foretinib (FTB). Mineralized nodules were visualized by Alizarin Red S staining (left), and calcium deposition was quantified by dye solubilization with cetylpyridinium chloride followed by absorbance measurement at 550 nm (right). Data represent mean ± SD from one of three independent experiments (triplicate per condition). * $p < 0.05$.

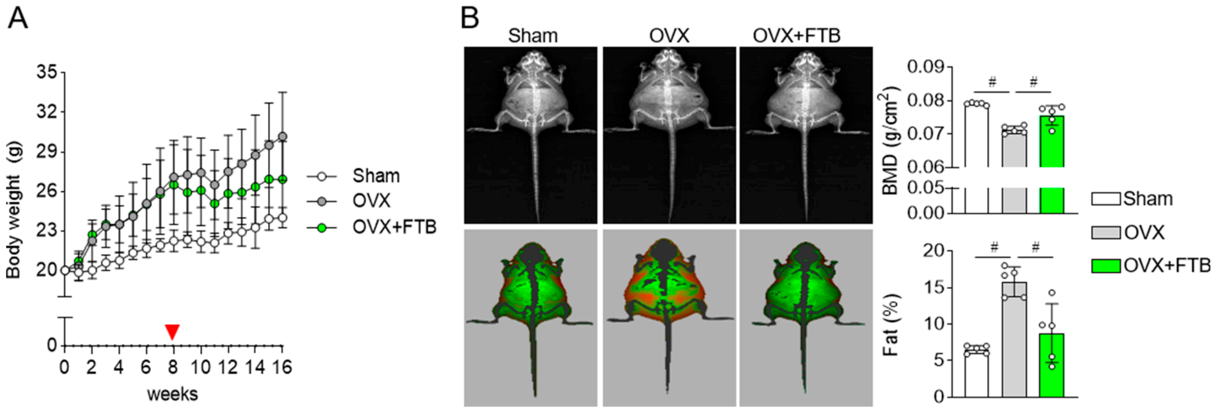

Figure S4. Body composition analysis. (**A**) Body weight profile. Ten-week-old female mice underwent sham surgery or ovariectomy (OVX) and were assigned to three groups: sham-operated (Sham), OVX, and OVX treated with foretinib (OVX+FTB). Eight weeks after surgery, foretinib (FTB; 0.315 mg/kg) or vehicle was administered intraperitoneally every 2 days for 8 weeks. Body weight was measured weekly for 16 weeks. The arrowhead indicates the onset of FTB administration. (**B**) DXA analysis. Mice were euthanized at 26 weeks of age, and bone mineral density (BMD) and fat mass were analyzed using a high-resolution DXA scanner. In the color composite image, fat and lean tissues are shown in red and green, respectively. Data are presented as mean ± SD (n = 5 per group). $^{\#}p < 0.01$.

Figure 1b (western blot images)

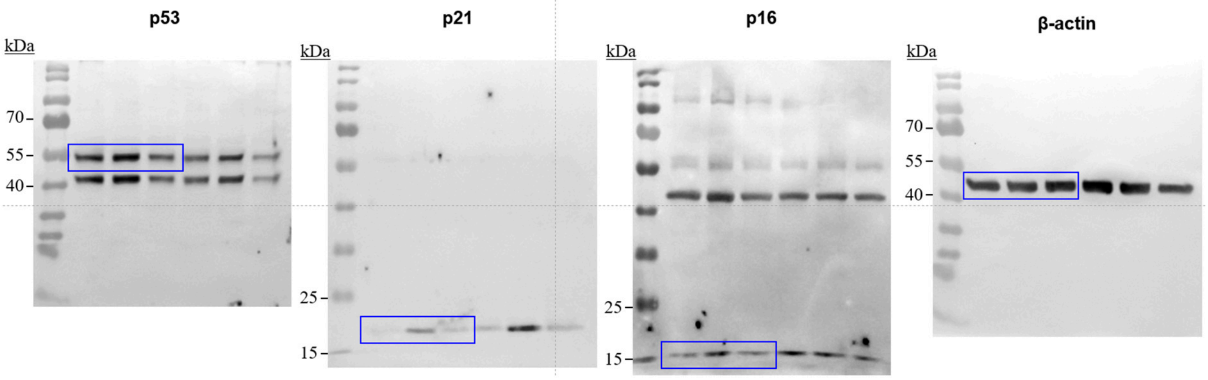

Figure 2b (western blot images)

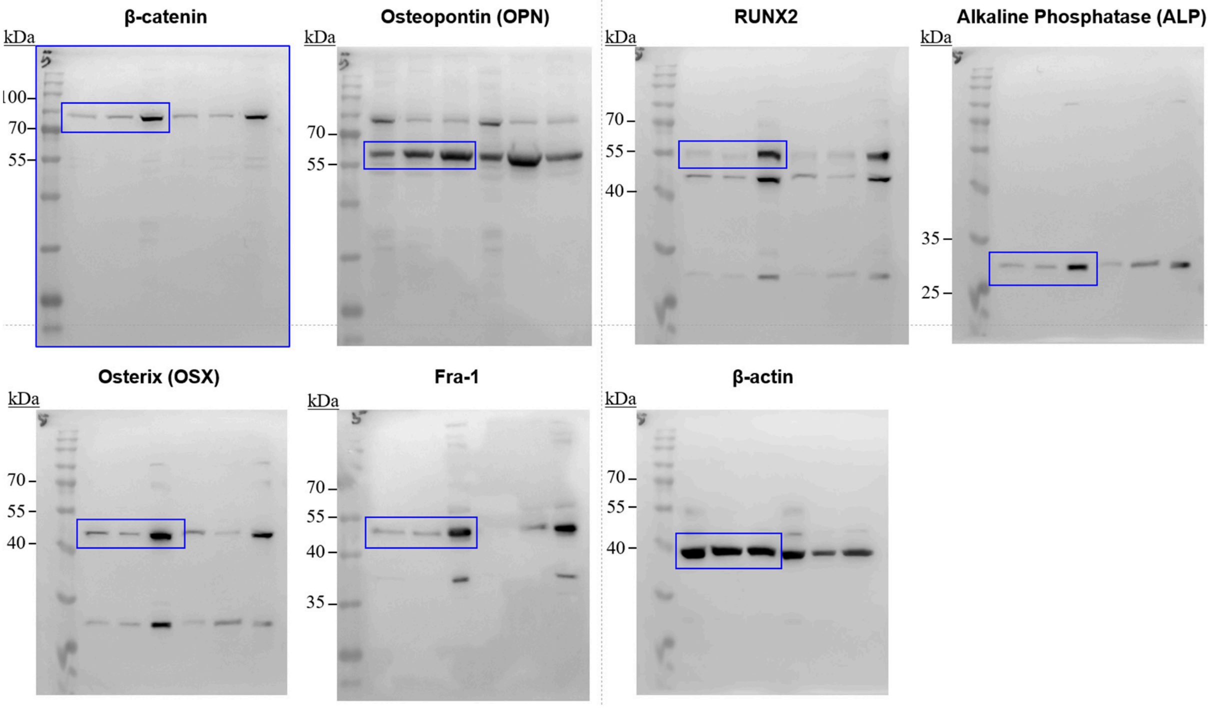

Supplement: Supplementary file 1 [file cells-14-01945-s001.zip › cells-3933693-supplementary.pdf]
